# Salvianolic Acid D Alleviates Cerebral Ischemia-Reperfusion Injury by Suppressing the Cytoplasmic Translocation and Release of HMGB1-Triggered NF-*κ*B Activation to Inhibit Inflammatory Response

**DOI:** 10.1155/2020/9049614

**Published:** 2020-01-22

**Authors:** Wen Zhang, Junke Song, Wan Li, Dewen Kong, Yu Liang, Xiaoyue Zhao, Guanhua Du

**Affiliations:** ^1^State Key Laboratory of Bioactive Substances and Functions of Natural Medicines, Institute of Materia Medica, Chinese Academy of Medical Sciences and Peking Union Medical College, Beijing 100050, China; ^2^Beijing Key Laboratory of Drug Target Identification and Drug Screening, Institute of Materia Medica, Chinese Academy of Medical Sciences and Peking Union Medical College, Beijing 100050, China

## Abstract

Inflammatory response participates in the overall pathophysiological process of stroke. It is a promising strategy to develop antistroke drugs targeting inflammation. This study is aimed at investigating the therapeutic effect and anti-inflammatory mechanism of salvianolic acid D (SalD) against cerebral ischemia/reperfusion (I/R) injury. A rat middle cerebral artery occlusion/reperfusion (MCAO/R) injury model was established, and an oxygen-glucose deprivation/reoxygenation (OGD/R) injury model was established in PC12 cells. Neurological deficit score, cerebral infarction, and edema were studied *in vivo*. Cell viability was achieved using the MTT method *in vitro*. The Bax, Bcl-2, cytochrome c, HMGB1, TLR4, TRAF6, NF-*κ*B p65, p-NF-*κ*B p65, and cleaved caspase-3 and -9 were tested via the Western blot method. Cytokines and cytokine mRNA, including TNF-*α*, IL-1*β*, and IL-6, were studied via ELISA and PCR methods. The translocation of HMGB1 and NF-*κ*B were studied by immunofluorescence assay. The HMGB1/NeuN, HMGB1/GFAP, and HMGB1/Iba1 double staining was carried out to observe the localization of HMGB1 in different cells. Results showed that SalD alleviated neurological impairment, decreased cerebral infarction, and reduced edema in I/R rats. SalD improved OGD/R-downregulated PC12 cell viability. SalD also promoted Bcl-2 expression and suppressed Bax, cytochrome c, and cleaved caspase-3 and -9 expression. SalD decreased the intensity of TLR4, MyD88, and TRAF6 proteins both *in vivo* and *in vitro*, and significantly inhibited the NF-*κ*B nuclear translocation induced by I/R and OGD/R. What's more, SalD inhibited HMGB1 cytoplasmic translocation in neurons, astrocytes, and microglia in both the cortex and hippocampus regions of I/R rats. In conclusion, SalD can alleviate I/R-induced cerebral injury in rats and increase the PC12 cell viability affected by OGD/R. The anti-inflammatory mechanism of SalD might result from the decreased nuclear-to-cytoplasmic translocation of HMGB1 and the inhibition on its downstream TLR4/MyD88/NF-*κ*B signaling.

## 1. Introduction

According to the epidemiological survey, stroke causes a high rate of human mortality and disability, and brings heavy burden to families and the society [1]. Ischemic stroke accounts for about 80%-90% of stroke [2–4]. One of the critical treatment methods for brain ischemia is to recover the blood supply effectively. Tissue plasminogen activator has been used clinically for the rescue of acute cerebral ischemia [5]. However, the severe hemorrhagic transformation injury followed by the recurrent flow of blood has limited its clinical application [6, 7]. The pathophysiological mechanism of cerebral ischemia and reperfusion injury is complex, and there are multiple mechanisms forming the complex regulatory network [8–10]. Inflammation participates in the overall pathophysiological process of stroke [11, 12]. The expression of inflammatory cytokines causes damage to brain tissue, destroys the blood-brain barrier, and induces the subsequent release of inflammatory mediators, which forms a vicious cycle of brain damage [13, 14]. Therefore, reducing the level of the inflammatory response is considered to be an essential method to treat cerebral ischemia injury.

High-mobility group box 1 (HMGB1) is a protein normally located inside the nucleus and involved in the construction and stabilization of nucleosomes and transcription of genes [15]. It is released into the cytoplasm and the extracellular space in large amounts during injury stimulation. HMGB1 release appears in the process of cell necrosis, apoptosis, and autophagy [16–18]. Once released, HMGB1 participates in and expands the inflammatory responses [19, 20]. Studies have shown that blood concentrations of HMGB1 are closely related to the severity and outcomes of stroke [21]. Inhibition of the cytoplasmic translocation and release of HMGB1 serves as a strategy for stroke treatment [22, 23]. Toll-like receptors (TLRs) link HMGB1 and inflammatory response [24]. The signal transduction pathways after the activation of TLRs are mainly divided into the myeloid differentiation protein 88- (MyD88-) dependent pathway and the non-MyD88-dependent pathway [25]. It has been confirmed by many experiments that TLR4 is relevant for the induction of inflammation. TLR4 participates in the whole pathological process of cerebral ischemia [26, 27]. After cerebral ischemia, NF-*κ*B can be found highly expressed in a variety of cells. As a critical link in the inflammatory response, NF-*κ*B can be activated by TLR4 [28, 29]. Activated NF-*κ*B induces the generation and release of various cytokines, which in turn stimulate the activation of NF-*κ*B. These cytokines are critical participants in the inflammatory cascade. They act as the downstream signals of NF-*κ*B, causing severe cerebral injury after stroke. By blocking the TLR4 recognition signal, the inflammation signal cannot be passed into the cell, thus inhibiting the activity of NF-*κ*B and inflammation. Therefore, the HMGB1/TLR4/NF-*κ*B inflammation cascade is an important pathway leading to the activation of the inflammatory response [30].

The active compounds extracted from traditional medicine might be important sources for anti-inflammatory agents [31–33]. Danshen, the dried root of *Salvia miltiorrhiza* Bge., is a commonly and widely used traditional medicine for the treatment of cardiovascular and cerebrovascular diseases, neurodegenerative diseases, and diabetic mellitus in clinical practice [34, 35]. As reported by chemistry research, effective compounds of Danshen are mainly summarized into two categories: lipophilic diterpenoids and water-soluble phenolic acids. The phenolic acids have been identified as natural cardiovascular and cerebrovascular protectants with a variety of pharmacological functions, such as anti-inflammation, antiplatelet aggregation, and antioxidation [36–42]. These phenolic acids have been accounted for most of the biological activities of Danshen, especially the beneficial impact on ischemia/reperfusion (I/R) damage [43]. However, in previous pharmacological research, comprehensive studies have been carried out mainly on common phenolic acids, such as salvianolic acid A (SalA) and salvianolic acid B (SalB), while other phenolic acids were less researched. Recently, with the development of modern analytical methods, new compounds can more easily be isolated from Danshen. Salvianolic acid D (SalD) is a potential bioactive compound extracted from it with increasing research interest [44]. Recent studies have shown that SalD processed good hydroxyl radical scavenging activity [45]. SalD also showed potential antiplatelet activity and peroxidase inhibition effect [46, 47]. And SalD has been identified as a valuable compound found in the Danhong injection used to relieve vascular endothelium impairment, which is used clinically for acute myocardial infarction [48]. SalD has also been screened as a potentially active component of the Shenxiong glucose injection, a pharmaceutical preparation for acute ischemic stroke in China [49]. However, more research attention should be paid to it in order to develop corresponding disease treatment drugs. So far, almost no anti-inflammatory effects of SalD have been reported. Therefore, it is of considerable significance to research the influence of SalD on the inflammatory response. In this paper, the therapeutic action of SalD against I/R injury in rats and PC12 cells was studied. The regulatory mechanism of SalD on HMGB1/TLR4/NF-*κ*B signaling was investigated.

## 2. Materials and Methods

### 2.1. Animals

Male Sprague-Dawley (SD) rats weighing 250 ± 10 g were supplied by Beijing Vital River Experimental Animal Co., Ltd. with the certificate no. SCXK2012-0001. SalD (CAS No.: 142998-47-8) was provided by the Institute of Materia Medica (CAMS & PUMC, Beijing, China). Rats were raised at 22 ± 2°C with food and water *ad libitum* and a half-day light-and-dark cycle. The study design and trial process were evaluated and confirmed by the Institutional Animal Care and Use Committee (CAMS & PUMC). Hard work was performed to ensure that rats experience minimal pain and discomfort. SD rats were randomly divided into 6 groups as follows: the sham operation group, the I/R group, the I/R+SalD (1, 3, and 15 mg/kg) groups, and the positive control nimodipine (20 mg/kg) group.

### 2.2. Cerebral Ischemia/Reperfusion Model

Isoflurane was used for anesthesia in rats. The anterior neck hairs were appropriately cut off. Then, an incision was operated on the neck midline. Muscle and fascia were isolated along the inner border of the sternocleidomastoid. The right common carotid artery (CCA), internal carotid artery (ICA), and external carotid artery (ECA) were separated. A nylon filament (diameter 0.2 mm) with a rounded tip was entered from CCA into ICA and reached into the middle cerebral artery (MCA). After ischemia for 1.5 h, the thin nylon wire was carefully removed to achieve reperfusion [50]. SalD was dissolved in normal saline. Rats were injected with SalD (1, 3, and 15 mg/kg) or nimodipine (20 mg/kg) intravenously at the onset of reperfusion and 12 h postreperfusion. The rats in the sham and I/R groups received an equal amount of normal saline. Other measurements and sample acquisition were performed 24 h after reperfusion.

### 2.3. Neurological Deficit Score

An investigator blinded to the rat groups assessed the neurological deficit score after 24 h I/R. According to the previously reported method, the scoring range was described as follows: no neurologic deficit (0 point), failure to fully extend the left forepaw (1 point), circling to the left (2 points), falling to the left (3 points), did not walk spontaneously, and had a depressed level of consciousness (4 points) [51]. Rats with 0 point (no brain damage) and 4 points (extremely severe cerebral injury) were excluded from the research.

### 2.4. Infarct Volume and Brain Water Content

Rats brains were harvested for the measurement of cerebral infarct volume and edema. For infarct volume determination, the brains were frozen and cut into 2 mm slices. Then, the slices were stained using 2% TTC (Sigma-Aldrich, MO, USA) and fixed using 4% paraformaldehyde. After that, the stained images were acquired by a digital camera. An investigator, blinded to the rat groups, outlined the infarct and the uninfarcted areas. To minimize the influence of edema on volume measurement, the adjusted infarct area was calculated using the entire contralateral hemisphere area minus the uninfarcted areas of the ischemic hemisphere [52, 53]. The adjusted infarct volume was acquired through integrating the adjusted infarct areas with the slice thickness. The result was exhibited as a percentage of the uninfarcted hemisphere. For the assessment of brain water content, brain wet weight was immediately acquired after harvesting. Then, the brain was dehydrated in a stove at 100°C for 48 h. This allowed us to get the dry weight. Brain water content (%) = (wet weight − dry weight)/wet weight × 100% [54].

### 2.5. Evans Blue Assay

Rats firstly received an intravenous injection of 4% Evans blue (0.2 mL/100 g, Sigma-Aldrich, MO, USA). After a period of Evans blue circulation, rats were perfused by 20 mL heparinized saline (10 U/mL). The brains were then removed and homogenized in 50% trichloroacetic acid solvent. The samples were centrifuged at 400 × g for 20 min, and the supernatant was acquired and tested at 620 nm. Evans blue content was calculated as ng/g tissue.

### 2.6. PC12 Cell Culture and Oxygen-Glucose Deprivation/Reoxygenation Model

PC12 cells (China Infrastructure of Cell Line Resources) were cultured with RPMI-1640 medium. The humidified atmosphere for cell culture contains 95% air and 5% CO_2_. The culture medium of PC12 cells contains 10% horse serum, 5% fetal bovine serum, 100 *μ*g/mL streptomycin, and 100 U/mL penicillin. Culture medium was renewed every 48 h. The culture flasks or plates were coated with polylysine 37°C for 2 h before being used. For the establishment of an OGD/R model, glucose-free RPMI-1640 medium and an incubator containing 94% N_2_, 5% CO_2_, and 1% O_2_ were used. Four hours later, full culture medium and a normal atmosphere were resupplied. Cell culture was continued with or without SalD, as well as the positive control N-acetyl-L-cysteine (NAC, Sigma-Aldrich, MO, USA) for 24 h. The experiment consisted of six groups: the normal control group (solvent only), the OGD/R stimulation model group (OGD/R), the SalD low dose group (OGD/R + 0.2 *μ*M SalD), the SalD middle dose group (OGD/R + 1 *μ*M SalD), the SalD high-dose group (OGD/R + 5 *μ*M SalD), and the positive control NAC group (OGD/R + 5 mM NAC).

### 2.7. MTT and LDH Assays

Cells were seeded and cultured at a density of 8 × 10^3^ cells per well. At the end of OGD/R stimulation and SalD or NAC treatment, cells in each well were incubated with 20 *μ*L MTT (5 *μ*g/*μ*L, Sigma-Aldrich, MO, USA) for 4 h. Then, cells in each well were added by 100 *μ*L DMSO. The plate was stirred for 15 min to dissolve the formazan product. The absorption was measured at 490 nm. Cell viability was shown as a percentage of the control group. For the LDH assay, at the end of OGD/R stimulation and SalD or NAC treatment, the cell supernatant was taken and the LDH release was measured according to the instructions of the LDH test kit (Beyotime Biotechnology, Shanghai, China).

### 2.8. ELISA Assay

ELISA kits were used to detect the levels of TNF-*α* (CSB-E11987r), IL-1*β* (CSB-E08055r), and IL-6 (CSB-E04640r, Cusabio Biotech, Wuhan, China) in the rat brain. Briefly, 0.1 g of the brain tissue was rinsed with 1 × PBS. Then, the brain tissue was homogenized in 1 mL of 1 × PBS and stored at -20°C overnight. After 2 freeze-thaw cycles, the homogenates were centrifuged for 5 min at 4°C (5000 × g). The supernatants were immediately transferred for measuring at 450 nm with a microplate spectrophotometer. The serum HMGB1 concentration was detected using the ELISA kit (CSB-E08224r, Cusabio Biotech Co., Ltd., Wuhan, China). For an in vitro assay, PC12 cells were incubated with different concentrations of SalD and OGD/R for 24 h. Then, the supernatant was removed and measured.

### 2.9. RT-qPCR Assay

An RT-qPCR assay was performed. The forward primers (5′-3′) for TNF-*α*, IL-1*β*, IL-6, and *β*-actin were GCTCCCTCTCATCAGTTCCA, GTACCTGTCCTGCGTGTTGA, and GTCAACTCCATCTGCCCTTC, and TGTCACCAACTGGGACGATA, respectively. The reverse primers (5′-3′) for TNF-*α*, IL-1*β*, IL-6, and *β*-actin were GCTTGGTGGTTTGCTACGAC, GGGAACTGGGCAGACTCAAA, TGTGGGTGGTATCCTCTGTG, and GGGGTGTTGAAGGTCTCAAA, respectively. The results of mRNA levels were expressed as a fold of untreated control.

### 2.10. Western Blot Analysis

The total proteins, nuclear proteins, and cytoplasmic proteins in brain tissues or PC12 cells were extracted and quantified using the BCA Protein Quantification Kit (Pulilai Gene Technology Co. Ltd., Beijing, China). The intensity of Bax, Bcl-2, cytochrome c, and cleaved caspase-3 and -9 (Cell Signaling Technology, MA, USA) was assessed from the cerebral cortex region. The intensity of NF-*κ*B p65, p-NF-*κ*B p65, and HMGB1 (Cell Signaling Technology, MA, USA) and the intensity of TLR4, TRAF6, and MyD88 (Abcam, Cambridge, UK) were assessed from both the cerebral cortex and hippocampus regions. The SDS buffer was then added to the protein and boiled for 10 min. The SDS-PAGE electrophoresis was carried out. Then, the proteins were transferred onto PVDF membranes and blocked with 5% BSA for 2 h at room temperature. Proteins were then reacted with different kinds of antibodies at 4°C overnight. After the incubation, the secondary antibodies were introduced and reacted for 1 h. The protein expressions of different samples were observed using the High Sensitivity Chemiluminescence Detection Kit (Kangwei Biological Company, Beijing, China).

### 2.11. Immunofluorescence Assay

The cortex and hippocampus regions of rats were applied for GFAP, Iba1, HMGB1/NeuN, HMGB1/GFAP, and HMGB1/Iba1 fluorescence. The slices were firstly incubated in 0.5% Triton X-100 (Sigma-Aldrich, MO, USA) solution for 20 min. Then, the slices were reacted with 10% goat serum for 30 min. After that, the antibodies of HMGB1, GFAP, and NeuN (Cell Signaling Technology, MA, USA) and the antibody of Iba1 (Abcam, Cambridge, UK) were employed and reacted overnight. After washing, the corresponding secondary antibodies of Alexa Fluor 488 or 594 (Thermo Fisher Scientific, Walthan, MA) were employed and reacted for 1 h. Then, DAPI was added (Sigma-Aldrich, MO, USA) and reacted for 10 min before being photographed. For TUNEL/NeuN fluorescence, terminal deoxynucleotidyl transferase (TdT) reaction solution was introduced specially and used according to the instruction of the TUNEL apoptosis detection kit (Roche, Mannheim, Germany). The expression and translocation of HMGB1 and p-NF-*κ*B p65 in PC12 cells were observed by cell immunofluorescence assay. Cells were firstly fixed with 4% paraformaldehyde for 15 min. Then, 0.3% Triton-100 was introduced and reacted for 10 min. After reacting with 5% BSA for 1 h, the primary antibodies of HMGB1 and p-NF-*κ*B p65 were introduced and reacted overnight at 4°C. After reacting, the secondary antibodies were introduced and reacted for 1.5 h. Finally, after Hoechst 33258 (Sigma-Aldrich, MO, USA) was applied, the fluorescence images were acquired under fluorescence microscopy.

### 2.12. Statistical Analysis

The measurement data were expressed as mean ± standard deviation (SD). The data were statistically analyzed using GraphPad Prism 7.0 software (GraphPad Software Inc., San Diego, CA, USA). The comparison was analyzed by one-way ANOVA or two-way ANOVA followed by Tukey's post hoc test. *P* < 0.05 was considered statistically significant.

## 3. Results

### 3.1. SalD Alleviated the Neurological Impairment, Decreased Cerebral Infarction, and Reduced Edema in I/R Rats

**Figure 1:**
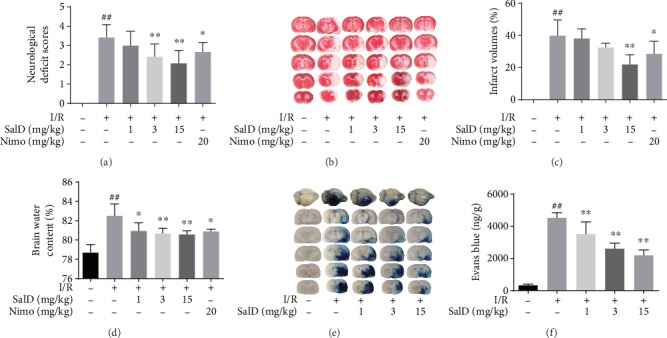
SalD alleviated the neurological impairment, decreased cerebral infarction, and reduced edema in I/R rats. (a) Neurological deficit score (*n* = 12). (b) Representative TTC-stained slices. (c) Infarct volume (n=6). (d) Brain water content (*n* = 6). (e) Representative Evans blue-stained brains and slices. (f) Evans blue content (*n* = 5). Data were expressed as means ± SD. ^##^*P* < 0.01 versus the sham operation group; ^∗^*P* < 0.05 and ^∗∗^*P* < 0.01 versus the MCAO/R group.

To work out whether SalD medication can promote the recovery of motor function in I/R rats, a neurological deficit test was conducted as previously described. I/R injury damaged the brain region that regulates body movement. The neurological impairment of the model rats was severe. SalD and nimodipine could effectively alleviate the neurological impairment in rats (Figure 1(a)). TTC staining revealed that no infarction was observed in the sham operation group, but significant infarction appeared in the ischemic cerebral hemisphere of the I/R rats (Figures 1(b) and 1(c), *P* < 0.01). The 15 mg/kg SalD and 20 mg/kg positive drug nimodipine significantly reduced the infarct volume in I/R rats (*P* < 0.01 and *P* < 0.05), respectively. I/R injury led to severe cytotoxic cerebral edema due to the inflammatory response and blood-brain barrier damage. As shown in Figure 1(d), obvious edema appeared in the ischemic hemisphere (*P* < 0.01), while the 1, 3, and 15 mg/kg SalD dose-dependently reduced cerebral edema (*P* < 0.05, *P* < 0.01, and *P* < 0.01). 20 mg/kg positive drug nimodipine also dramatically alleviated the degree of I/R-induced cerebral edema. The content of Evans blue in I/R rats was notably higher (Figures 1(e) and 1(f), *P* < 0.01), indicating that the blood-brain barrier was severely damaged. The content of Evans blue in SalD treatment groups (1, 3, and 15 mg/kg) was significantly downregulated (*P* < 0.01), and the blood-brain barrier injury was alleviated. The results suggested that SalD could inhibit the blood-brain barrier injury and reduce its permeability in I/R rats.

### 3.2. SalD Inhibited Apoptosis Induced by I/R Injury in Rats

**Figure 2:**
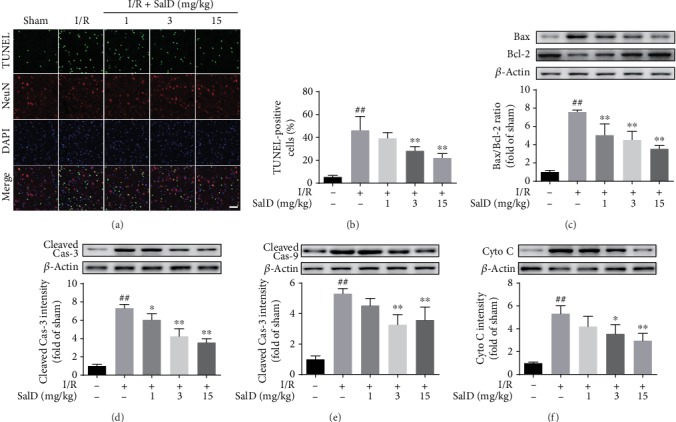
SalD inhibited apoptosis induced by I/R injury in rats. (a) Representative TUNEL/NeuN immunofluorescence photograph. (b) The percent of TUNEL-positive cells. (c) The relative Bax/Bcl-2 ratio (*n* = 4). (d) The relative intensity of cleaved caspase-3 (*n* = 4). (e) The relative intensity of cleaved caspase-9 (*n* = 4). (f) The relative intensity of cytochrome c (*n* = 4). Proteins were acquired from the cerebral cortex region and assayed by the Western blot method. The intensity was normalized to *β*-actin. Data were expressed as means ± SD. ^##^*P* < 0.01 versus the sham operation group; ^∗^*P* < 0.05 and ^∗∗^*P* < 0.01 versus the MCAO/R group. Scale bar = 50 *μ*m.

The DNA damage caused by I/R was identified by TUNEL assay. Only a small amount of TUNEL fluorescence was observed in the sham rats, and strong TUNEL green fluorescence was found in the I/R rats with a higher apoptotic rate (Figure 2(a), *P* < 0.01). Three and 15 mg/kg SalD alleviated DNA damage and decreased the percentage of TUNEL-positive cells notably (*P* < 0.01, Figure 2(b)). As shown by the Western blot method in Figures 2(c) and 2(f), the intensity of Bax and cytochrome c in the injured brain area were elevated forcefully, and the intensity of Bcl-2 was downregulated. Three and 15 mg/kg SalD significantly reduced the intensity of Bax and cytochrome c and elevated the intensity of Bcl-2. The levels of cleaved caspase-3 and -9 were significantly upregulated in the I/R-injured rat brain (*P* < 0.01), while SalD treatment significantly obstructed the activation of caspase-3 and -9 (Figures 2(d) and 2(e)).

### 3.3. SalD Reduced the Inflammatory Response Induced by I/R Injury in Rats

**Figure 3:**
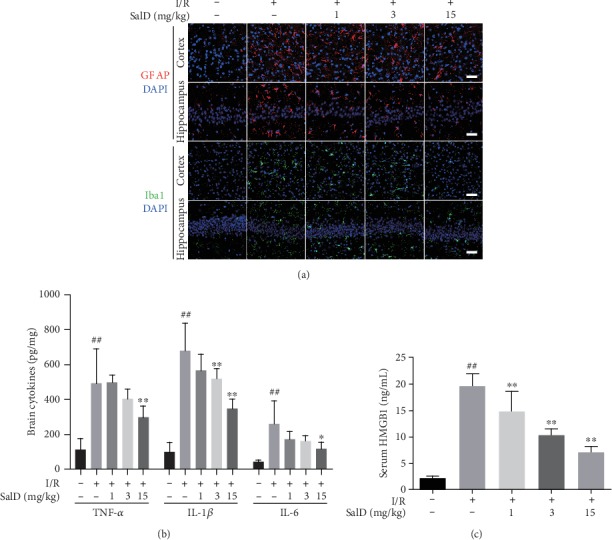
SalD reduced the inflammatory response induced by I/R injury in rats. (a) SalD inhibited the astrocytes and microglia activation in I/R rats. Representative figures showing GFAP and Iba1 staining. (b) SalD downregulated the generation of TNF-*α*, IL-1*β*, and IL-6 in I/R rats. (c) SalD attenuated the upregulated release of serum HMGB1 in I/R rats. Data are expressed as means ± SD. ^##^*P* < 0.01 versus the sham operation group; ^∗^*P* < 0.05 and ^∗∗^*P* < 0.01 versus the MCAO/R group. Scale bar = 50 *μ*m.

Astrocytes and microglia in brain tissue were labeled with GFAP and Iba1 staining, respectively. The number of microglia and astrocytes in the injured brain area of the model rats was notably higher than the sham rats. SalD treatment effectively inhibited the activation of microglia and astrocytes in brain tissue (Figure 3(a)). The contents of TNF-*α*, IL-1*β*, and IL-6 in brain tissue were investigated by ELISA assay. I/R damage increased the generation of TNF-*α*, IL-1*β*, and IL-6 (*P* < 0.01). SalD treatment significantly decreased the generation of cytokines in brain tissue (Figure 3(b)). SalD also attenuated the upregulated release of serum HMGB1 dose-dependently (Figure 3(c)).

### 3.4. SalD Increased PC12 Cell Viability and Inhibited LDH Release

**Figure 4:**
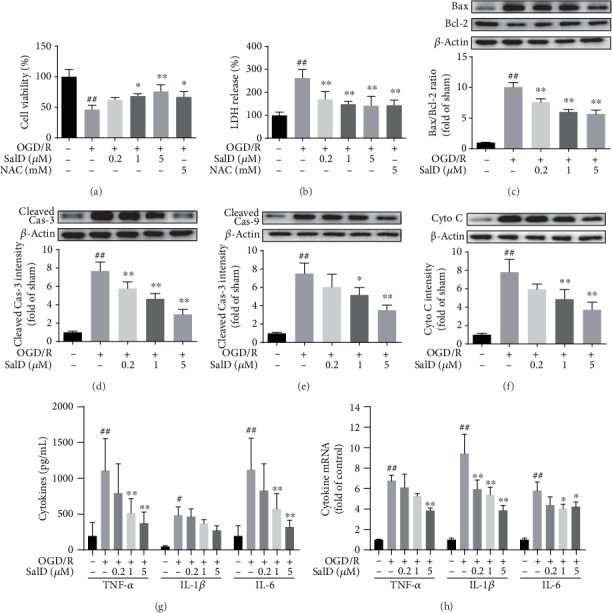
SalD increased cell viability, reduced LDH release, and inhibited apoptosis and the inflammatory response induced by OGD/R injury in PC12 cells. (a) Cell viability (*n* = 4). (b) LDH release (*n* = 4). (c) The relative Bax/Bcl-2 ratio (*n* = 4). (d) The relative intensity of cleaved caspase-3 (*n* = 4). (e) The relative intensity of cleaved caspase-9 (*n* = 4). (f) The relative intensity of cytochrome c (*n* = 4). (g) The generation of TNF-*α*, IL-1*β*, and IL-6 cytokines (*n* = 6). (h) The transcription of TNF-*α*, IL-1*β*, and IL-6 mRNA (*n* = 4). Proteins were acquired from PC12 cells and assayed by the Western blot method. The intensity was normalized to *β*-actin. Data were expressed as means ± SD. ^##^*P* < 0.01 versus the control group; ^∗^*P* < 0.05 and ^∗∗^*P* < 0.01 versus the OGD/R group.

MTT assay was operated to observe the impact of SalD on the cell viability downregulated by OGD/R.  Figure 4(a) reveals that OGD/R extremely lowered PC12 cell viability (*P* < 0.01), while SalD (1 and 5 *μ*M) raised the cell viability productively (*P* < 0.05 and *P* < 0.01). The positive control NAC also adequately raised the cell viability (*P* < 0.05). MTT assay showed that SalD could reverse the damage caused by OGD/R to PC12 cells and significantly improve the cell viability.  Figure 4(b) shows that the LDH release in PC12 cells was significantly increased by OGD/R injury, and SalD (0.2, 1, and 5 *μ*M) concentration-dependently decreased LDH release in comparison with the OGD/R-damaged cells (*P* < 0.01). The positive control NAC also lowered LDH release effectively (*P* < 0.01).

### 3.5. SalD Inhibited Apoptosis Induced by OGD/R Injury in PC12 Cells

Western blot was performed to reveal the relative intensity of apoptosis-related proteins *in vitro*.  Figure 4(c) shows that the Bax/Bcl-2 ratio in OGD/R-damaged cells was extremely higher than that in untreated cells. SalD promoted the expression of Bcl-2 and inhibited the overexpression of Bax, thereby promoting the recovery of Bax/Bcl-2 balance and reducing OGD/R-induced neuronal apoptosis in PC12 cells.  Figure 4(d) shows that OGD/R impairment extremely increased the levels of cleaved caspase-3 (*P* < 0.01), while SalD (0.2, 1, and 5 *μ*M) concentration-dependently decreased the levels of cleaved caspase-3 (*P* < 0.01).  Figure 4(e) shows that OGD/R stimulation considerably induced the production of cleaved caspase-9 (*P* < 0.01), while SalD (1 and 5 *μ*M) restrained the protein induction process effectively (*P* < 0.05 and *P* < 0.01). Cytochrome c is one of the most critical proteins in the mitochondrial apoptotic pathway.  Figure 4(f) reveals that OGD/R damage enhanced the release of cytochrome c significantly, while SalD (1 and 5 *μ*M) notably downregulated the release of cytochrome c (*P* < 0.01).

### 3.6. SalD Inhibited the Production of TNF-*α*, IL-1*β*, and IL-6 Induced by OGD/R Injury

The formation of TNF-*α*, IL-1*β*, and IL-6 by PC12 cells was assessed through ELISA kits. Data demonstrated that OGD/R intervention notably upregulated the mRNA levels and the generation of TNF-*α*, IL-1*β*, and IL-6 by PC12 cells (*P* < 0.01, Figures 4(g) and 4(h)). SalD significantly reduced the transcription of TNF-*α* mRNA (Figure 4(g)) and decreased TNF-*α* production (Figure 4(h)). SalD also dose-dependently reduced the transcription of IL-1*β* mRNA in OGD/R-injured PC12 cells (Figure 4(g)). What more, SalD (1 and 5 *μ*M) significantly decreased IL-6 mRNA levels (*P* < 0.01, Figure 4(g)) and downregulated the generation of IL-6 in PC12 cells (*P* < 0.01, Figure 4(h)).

### 3.7. SalD Inhibited HMGB1-Triggered NF-*κ*B Activation in OGD/R-Injured PC12 Cells

**Figure 5:**
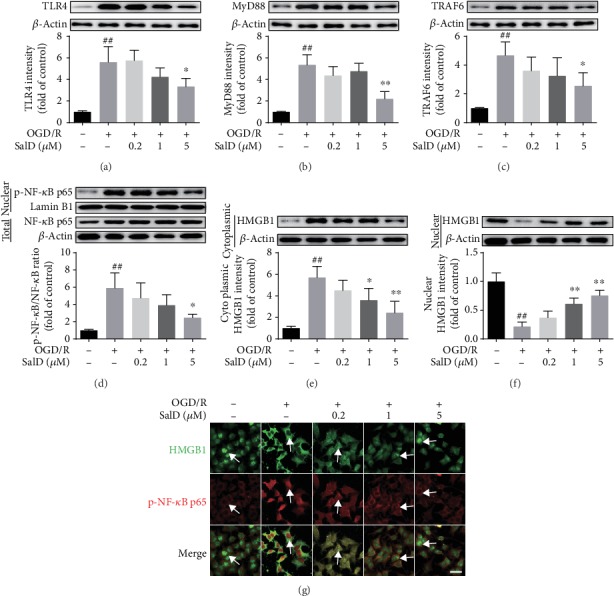
SalD inhibited HMGB1-triggered NF-*κ*B activation in OGD/R-injured PC12 cells. (a) The relative intensity of TLR4 (*n* = 4). (b) The relative intensity of MyD88 (*n* = 4). (c) The relative intensity of TRAF6 (*n* = 4). (d) The relative p-NF-*κ*B p65/NF-*κ*B p65 ratio (*n* = 4). (e) The relative intensity of cytoplasmic HMGB1 (*n* = 4). (f) The relative intensity of nuclear HMGB1 (*n* = 4). (g) The immunofluorescence image of HMGB1 and p-NF-*κ*B p65 double staining in PC12 cells. Proteins were acquired from PC12 cells and assayed by the Western blot method. Nuclear proteins were normalized to the intensity of Lamin B1, while other proteins were normalized to the intensity of *β*-actin. Data were expressed as means ± SD. ^##^*P* < 0.01 versus the control group; ^∗^*P* < 0.05 and ^∗∗^*P* < 0.01 versus the OGD/R group. Scale bar = 20 *μ*m.

Western blot was employed to identify the intensity of HMGB1, TLR4, MyD88, TRAF6, and NF-*κ*B proteins.  Figure 5(a) shows that the levels of TLR4 were notably elevated in the OGD/R group (*P* < 0.01), while SalD (5 *μ*M) significantly inhibited the TLR4 activation resulting from OGD/R damage (*P* < 0.05).  Figure 5(b) shows that the levels of MyD88 were extremely upregulated in the OGD/R group (*P* < 0.01), while SalD (5 *μ*M) inhibited the OGD/R-induced MyD88 upregulation significantly (*P* < 0.01).  Figure 5(c) demonstrates that the intensity of TRAF6 was significantly strengthened (*P* < 0.01) in the OGD/R group, while SalD (5 *μ*M) inhibited OGD/R-induced expression of TRAF6 significantly (*P* < 0.05).  Figure 5(d) presents the nuclear intensity of the stimulated pattern of p-NF-*κ*B p65 that was increased dramatically by OGD/R injury (*P* < 0.01), while SalD (5 *μ*M) suppressed the OGD/R-mediated NF-*κ*B activation significantly (*P* < 0.05).  Figure 5(e) shows that the intensity of cytoplasmic HMGB1 was increased dramatically by the OGD/R stimulation (*P* < 0.01), while SalD (1 and 5 *μ*M) prevented the OGD/R-mediated HMGB1 cytoplasmic translocation effectively (*P* < 0.05 and *P* < 0.01).  Figure 5(f) shows that the intensity of nuclear HMGB1 was decreased considerably in OGD/R rats (*P* < 0.01), while SalD (1 and 5 *μ*M) downregulated the OGD/R-induced HMGB1 release from the nucleus significantly (*P* < 0.01). Cell immunofluorescence assay was employed to observe the regulation of SalD on the translocation of HMGB1 and p-NF-*κ*B p65. Results showed that SalD significantly inhibited the cytoplasmic translocation of HMGB1, as well as the nuclear translocation of p-NF-*κ*B p65 induced by OGD/R (Figure 5(g)).

### 3.8. SalD Inhibited HMGB1 Cytoplasmic Translocation and NF-*κ*B Nuclear Translocation in Cortex and Hippocampus of I/R Rats

**Figure 6:**
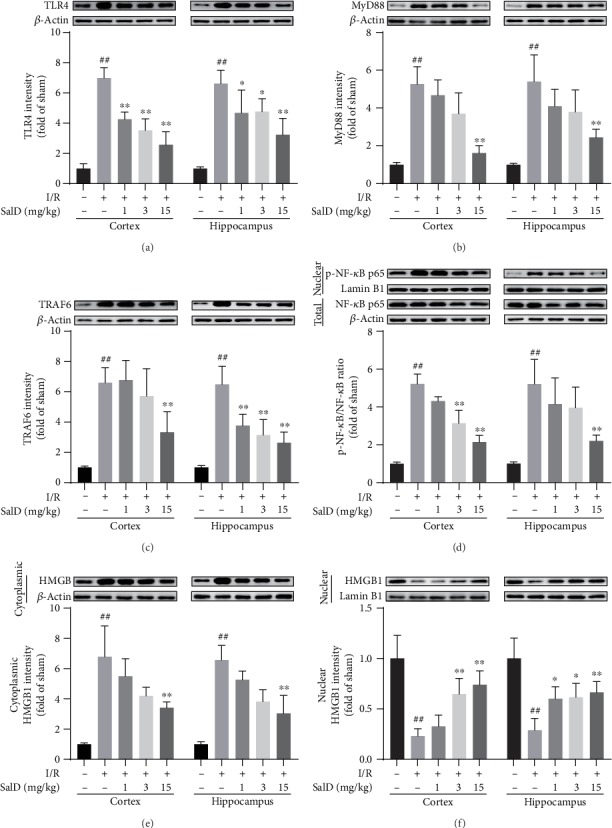
SalD inhibited HMGB1 cytoplasmic translocation and NF-*κ*B nuclear translocation in the cortex and hippocampus of I/R rats. (a) The relative intensity of TLR4 (*n* = 4). (b) The relative intensity of MyD88 (*n* = 4). (c) The relative intensity of TRAF6 (*n* = 4). (d) The relative p-NF-*κ*B p65/NF-*κ*B p65 ratio (*n* = 4). (e) The relative intensity of cytoplasmic HMGB1 (*n* = 4). (f) The relative intensity of nuclear HMGB1 (*n* = 4). Protein samples were acquired from the cortex or hippocampus region and assayed by the Western blot method. Nuclear proteins were normalized to the intensity of Lamin B1, while other proteins were normalized to the intensity of *β*-actin. Data were expressed as means ± SD. ^##^*P* < 0.01 versus the sham operation group; ^∗^*P* < 0.05 and ^∗∗^*P* < 0.01 versus the MCAO/R group.

In comparison with the sham rats, the intensity of TLR4 in the cerebral cortex and hippocampus of the I/R-damaged rats was significantly increased (Figure 6(a), *P* < 0.01). The SalD treatment groups (1, 3, and 15 mg/kg) dose-dependently reduced TLR4 levels. In comparison with the sham rats, the expression of Myd88 and TRAF6 proteins was enhanced in the model group (*P* < 0.01, Figures 6(b) and 6(c)). The SalD medication groups significantly reduced the protein expression of Myd88 and TRAF6. The NF-*κ*B pathway was greatly activated in I/R-impaired rats. The nuclear phosphorylation degree of NF-*κ*B p65 was significantly increased in the cortex region and the hippocampus region (Figure 6(d), *P* < 0.01). Compared with the model group, the SalD treatment groups remarkably lowered the nuclear content of p-NF-*κ*B p65 both in the cortex and hippocampus. HMGB1 cytoplasmic translocation was extremely elevated in the I/R-impaired group (*P* < 0.01, Figure 6(e)). SalD treatment inhibited the cytoplasmic translocation of HMGB1 and restored its nuclear staining both in the cortex region and the hippocampus region (Figure 6(f)).

### 3.9. SalD Inhibited HMGB1 Cytoplasmic Translocation in Neurons, Astrocytes, and Microglial Cells in the Cortex and Hippocampus of I/R Rats

**Figure 7:**
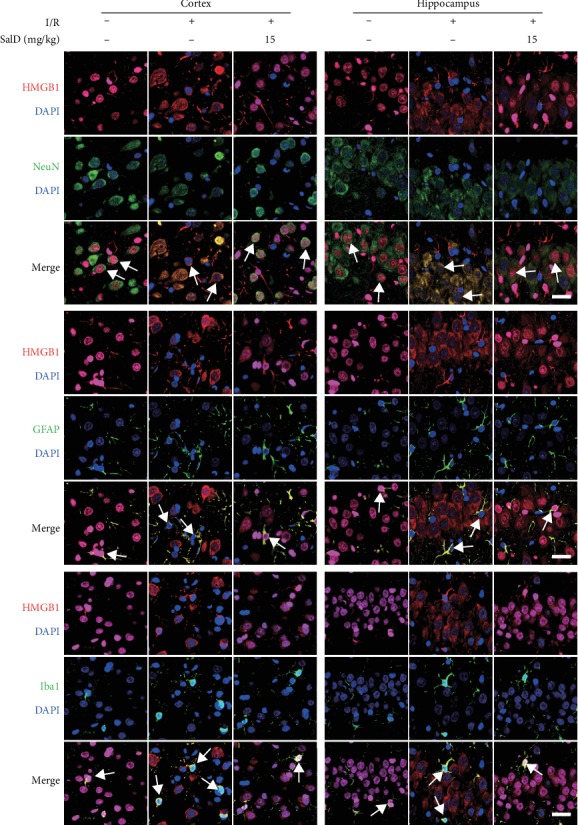
SalD inhibited HMGB1 cytoplasmic translocation in neurons, astrocytes, and microglial cells in the cortex and hippocampus of I/R rats. The immunofluorescent assay was conducted using the HMGB1/NeuN, HMGB1/GFAP, and HMGB1/Iba1 staining. Scale bar = 25 *μ*m.

The immunofluorescent assay was conducted using HMGB1/NeuN, HMGB1/GFAP, and HMGB1/Iba1 staining. In the sham group, HMGB1 remained in the nucleus of neurons, astrocytes, and microglial cells (Figure 7). But in the I/R model group, HMGB1 was translocated out of the nucleus to the cytoplasm. SalD (15 mg/kg) inhibited HMGB1 cytoplasmic translocation in neurons, astrocytes, and microglial cells both in the cortex and hippocampus of I/R rats.

## 4. Discussion and Conclusion

A variety of mechanisms have been connected with the process of cerebral I/R injury, especially the inflammatory response [12]. The overexpression of cytokines induced by inflammatory response is a classic pathology feature of I/R injury [55]. In this paper, SD rats were subjected to I/R damage, and PC12 cells were subjected to OGD/R injury. SalD alleviated neurological impairment, decreased cerebral infarction, and reduced edema in I/R rats. SalD also reduced Evans blue extravasation and protected the blood-brain barrier of I/R rats. In PC12 cells, SalD suppressed the cell death caused by OGD/R and improved cell viability. SalD also induced Bcl-2 expression and inhibited Bax, cytochrome c, and cleaved caspase-3 and -9 expression to alleviate apoptosis injury both *in vivo* and *in vitro*.

Danshen, a traditional medicine, has a long application history of treating diseases. The phenolic compounds in Danshen have been investigated for the therapeutic effects on cerebrovascular diseases, cardiovascular diseases, neurodegenerative diseases, diabetic mellitus, etc. [34, 35]. SalA and SalB are the two most studied phenolic compounds in Danshen. SalA and SalB have been observed to possess a defensive effect against pathological disorders owing to their antioxidative activities [36, 56, 57]. SalA and SalB also exerted the therapeutic effects via the inhibition of inflammatory response [58–61]. What's more, according to the recent pharmacological studies, SalA and SalB have shown potential therapeutic approaches against ischemic diseases. SalA alleviated I/R-induced rat brain damage partially via the suppression of FoxO3a/Bim signaling and the downregulation of MMP-9 activity [39, 62, 63]. SalA also showed antimyocardial I/R damage effects as a consequence of the downregulation of platelet activation and inflammation in rats [41, 64]. SalA protected against I/R-induced liver injury via inhibiting TLR4-mediated inflammation in rats [65]. And through the activation of Nrf2 and SIRT1 pathways, SalB attenuated the brain oxidative stress, inflammation response, and apoptosis in stroke rats [66–68]. The inhibition of TLR4/MyD88 signaling also contributed to the protective effect of SalB against I/R-induced rat cerebral inflammatory response [69]. In addition, SalB inhibited the platelet activation and neuroinflammation induced by cerebral ischemic injury in rats [70]. SalB also protects against nonalcoholic fatty liver injury via the inhibition of HMGB1 release [71]. What's more, SalA and SalB exerted therapeutic effects on neurodegenerative diseases. The mechanism of their antineurodegenerative disease effect involved the inhibition of A*β* aggregation, neurotoxicity, neuroinflammation, and oxidative stress [72–74]. Rosmarinic acid is another structure-similar compound of SalD. Rosmarinic acid has also been identified as a potential anti-inflammatory agent. It mitigated LPS-mediated neuroinflammation via the suppression of TLR4/NF-*κ*B signaling and the activation of NLRP3 inflammasome [75]. It also attenuated LPS-induced microglial cell activation and the release of inflammatory cytokines [76]. Studies showed that rosmarinic acid elicited neuroprotection effects in animal ischemic stroke models through the initiation of Nrf2/HO-1 signaling and the antiapoptotic effect [77, 78]. Rosmarinic acid also ameliorated hypoxia/ischemia-induced cognitive deficits and promoted remyelination in rats [79]. As a structural analogue of the compounds mentioned above, the research on the pharmacological activity of salvianolic acid D is of great significance, especially in the treatment of ischemia/reperfusion injury.

HMGB1 is a very conservative nuclear protein, released by stimulated monocytes, macrophages, or other damaged cells [80, 81]. The cytoplasmic translocation and release of HMGB1 will stimulate the immune cells and result in severe inflammatory response [82]. HMGB1 participates in inflammatory reactions primarily through signal transduction, and then interacts with cytokines, inflammatory mediators, and adhesion molecules [83, 84]. The currently known HMGB1 receptors mainly include TLRs and RAGE, among which TLR4 plays the most crucial role in I/R injury. After binding with TLR4, HMGB1 interacts with myeloid differentiation factor 88 (MyD88), thereby promoting the activation of downstream signal NF-*κ*B, thus aggravating inflammatory response and cerebral ischemia injury [85]. Many studies have found that HMGB1 could be passively released during ischemia and is an early mediator of inflammation after I/R injury. More importantly, clinical data suggested that elevated circulating HMGB1 levels were associated with poor neurological outcomes [86]. Our results showed that SalD significantly inhibited the OGD/R-induced HMGB1 release into cytoplasm in PC12 cells. Immunofluorescence data showed that the nuclear level of HMGB1 was negatively correlated with the nuclear level of p-NF-*κ*B p65. And SalD treatment significantly inhibited HMGB1 cytoplasmic translocation in neurons, astrocytes, and microglial cells. The translocation inhibition of HMGB1 was found in both the cerebral cortex and hippocampus of I/R rats. What's more, the serum concentration of HMGB1 in rats was also effectively downregulated by SalD treatment.

TLR4 in neurons, astrocytes, and microglia cells of the central nervous system contributes to I/R-induced inflammatory response. Several anti-inflammatory treatments against the activation of TLR4 have been shown to alleviate the damage caused by I/R injury [26, 87, 88]. In this experiment, we found that TLR4 was increased in the brain after reperfusion injury. Evidence has shown that HMGB1 induces inflammatory reaction primarily through direct interactions with TLR4. Activated TLR4 binds with MyD88, then attaches to IRAK and phosphorylates it. Phosphorylated IRAK binds with TRAF6 and activates it. TRAF6 further enables TAK1 to activate IKKs. Activated IKKs then phosphorylates I*κ*B, which are further ubiquitinated and degraded by ubiquitin ligase complex, releasing the active state of NF-*κ*B to initiate the transcription and translation of related genes [89, 90]. In our study, the expression of TLR, MyD88, and TRAF6 proteins was enhanced in the model injury group. The SalD treatment significantly reduced the intensity of TLR4, Myd88, and TRAF6 both in the cerebral cortex and hippocampus of I/R rats.

NF-*κ*B is an essential transcriptional regulator, participating in diseases associated with inflammation [91]. In the normal state, cytoplasmic NF-*κ*B exists in an inactive complex form binding to I*κ*B. Different signaling pathways, such as HMGB1/TLR4/MyD88, can phosphorylate I*κ*B, releasing the active form of NF-*κ*B. Activated NF-*κ*B promotes the production and release of various cytokines, including IL-1*β*, TNF-*α*, and IL-6. NF-*κ*B plays vital roles in I/R-induced cerebral inflammatory response, primarily through the regulation on its downstream cytokines. In our study, the nuclear translocation inhibition of p-NF-*κ*B p65 was found in both the cerebral cortex and hippocampus of I/R rats. Cytokines and cytokine mRNA, including TNF-*α*, IL-1*β*, and IL-6, were notably downregulated by SalD treatment. Our data indicated that SalD decreased the nuclear translocation of p-NF-*κ*B p65 and effectively inhibited the production of inflammation-related cytokines.

In summary, SalD can alleviate I/R-induced cerebral injury in rats and increase PC12 cell viability affected by OGD/R. SalD decreased the intensity of TLR4, MyD88, and TRAF6 both *in vivo* and *in vitro*, and significantly inhibited NF-*κ*B nuclear translocation. The anti-inflammatory mechanism of SalD might result from the decreased nuclear-to-cytoplasmic translocation of HMGB1 and the inhibition on its downstream TLR4/MyD88/NF-*κ*B signaling pathway.

## Data Availability

The data used to support the findings of this study are available from the corresponding author upon request.
